# The effect of HA380 blood adsorption on patients with acute infective endocarditis undergoing cardiac surgery: a retrospective study

**DOI:** 10.3389/fcvm.2025.1512619

**Published:** 2025-03-11

**Authors:** Xiao Jiefei, Cao Lu, Shi Han, Shi Yongxu, Mo Shaoyan, Qin Kai, Li Yonghua, Zhu Yanling, Jiang Yumei, Rong Jian

**Affiliations:** ^1^ Department of Extracorporeal Circulation, First Affiliated Hospital of Sun Yat-sen University, Guangzhou, China; ^2^ NHC Key Laboratory of Assisted Circulation and Vascular Diseases, Sun Yat-sen University, Guangzhou, China

**Keywords:** infective endocarditis, HA380, blood adsorption, postoperative sepsis, cardiopulmonary bypass

## Abstract

**Introduction:**

Sepsis is a major cause of ICU admission and mortality in patients with infective endocarditis patients. This study aimed to explore the effect of intraoperative HA380 blood adsorption on surgical outcomes in infective endocarditis patients, given its ability to adsorb inflammatory factors.

**Methods:**

We retrospectively analyzed the clinical data of patients who underwent surgical treatment for infective endocarditis at our hospital. After propensity score matching, eligible patients were matched in a 1:1 ratio between HA380 users and non-users. The primary endpoint was the incidence of postoperative sepsis, while secondary outcomes included ICU stay, postoperative hospital stay, and the need for CRRT, IABP, and ECMO therapies. Laboratory results were compared at 24, 48, and 72 h postoperatively.

**Results:**

A total of 148 patients were included in the analysis. After 1:1 matching, 39 pairs were further analyzed. There was no significant difference in the incidence of postoperative sepsis (20.5% vs. 15.4%, *p* = 0.724). However, HA380 patients had a significantly shorter postoperative hospital stay (21.2 vs. 28.1 days, *p* = 0.014), with no differences observed in the use of CRRT, IABP, or ECMO. Laboratory results showed that HA380 patients had significantly lower fibrinogen levels and a higher albumin-to-fibrinogen ratio.

**Discussion:**

This study did not demonstrate a reduced risk of postoperative sepsis with HA380 blood adsorption. Although the HA380 group had a shorter postoperative hospital stay, lower fibrinogen levels, and a higher albumin-to-fibrinogen ratio, the overall effectiveness of HA380 requires further investigation.

## 1 Background

The incidence of infective endocarditis varies by region, ranging from 2 to 10 cases per 100,000 individuals (1–3). Despite significant advancements in diagnosis, treatment, and perioperative management, the in-hospital mortality rate remains high, exceeding 20% (4–7). Surgical intervention is the primary approach for valve reconstruction in infective endocarditis, and in-hospital mortality is closely associated with sepsis-induced multiple organ dysfunction syndrome (MODS) (8, 9).Sepsis-related inflammatory factors are believed to trigger an excessive systemic inflammatory response (SIRS), which can lead to MODS (10). Therefore, reducing inflammatory factors through intraoperative blood adsorption is considered a promising strategy. While the HA380 adsorption filter has demonstrated efficacy *in vitro* (4, 11–14), its *in vivo* effectiveness remains a subject of debate. This study aims to evaluate the impact of HA380 blood adsorption on surgical outcomes in patients with infective endocarditis.

## 2 Materials and methods

### 2.1 Patients

This single-center, retrospective observational study analyzed data from 148 patients with infective endocarditis who underwent cardiac surgery at our hospital between January 1, 2019, and March 1, 2022.

Inclusion criteria: Patients with acute infective endocarditis undergoing valve reconstruction surgery were included.

Exclusion criteria: (1) Inability to undergo valve reconstruction surgery. (2) Incomplete clinical information. (3) Postoperative pathological results indicating non-bacterial endocarditis, such as marantic endocarditis related to malignancy or Libman-Sacks endocarditis associated with systemic lupus erythematosus (15).

### 2.2 Outcome

The primary outcome of this study was the incidence of postoperative sepsis, with sepsis diagnosed based on the third international consensus definition (16, 17). Sepsis is defined as life-threatening organ dysfunction caused by a dysregulated host response to infection. Patients with an increase in Sequential Organ Failure Assessment (SOFA) score of ≥2 and suspected or proven infection were considered to have sepsis.

Secondary outcomes included length of stay, postoperative hospital stay, use of continuous renal replacement therapy (CRRT), intra-aortic balloon pump (IABP) and extracorporeal membrane oxygenation (ECMO).

### 2.3 Data collection

General clinical data of patients were collected, such as patient gender, age, medical history, preoperative laboratory test results. Intraoperative data included surgical approach, intraoperative transfusion and blood product usage, and cardiopulmonary bypass-related data (aortic cross-clamp time, hypothermic time). Postoperative data included the occurrence of sepsis, length of hospital stay, ICU stay, major postoperative complications, use of CRRT or ECMO, and postoperative continuous laboratory test results.

### 2.4 Use of HA380

The HA380 blood perfusion device was integrated with the cardiopulmonary bypass (CPB) circuit for blood perfusion. Venous blood initially entered the reservoir and subsequently flowed into the oxygenator, driven by the pump. After oxygenation, the majority of oxygenated blood was directed into the arterial circulation for perfusion, with approximately 700 ml/min (14%–18%) undergoing blood filtration through the HA380 blood perfusion device. The filtered blood was then returned to the reservoir and mixed with venous blood. No additional equipment was required for this process.

### 2.5 Data analysis

Statistical analysis was performed using SPSS software, and propensity score matching (PSM) analysis was conducted using the R language. The propensity score (PS) was derived from a multivariate logistic regression model that estimated the group affiliation (HA380 vs. non-HA380) based on variables such as gender, age, medical history, preoperative laboratory test results, and more. The nearest neighbor algorithm was employed for 1:1 PSM to minimize potential confounding effects and achieve covariate balance between the groups. Given the high mortality rate associated with infective endocarditis but its relatively low incidence, a caliper was not applied to maximize the use of patients who received HA380. To After matching, comparisons between groups were made using the paired *t*-test or rank sum test for continuous variables and the McNemar test for categorical variables. A *p*-value of <0.05 was considered statistically significant.

### 2.6 Ethics

This study was designed in accordance with the principles outlined in the the Helsinki Declaration and complied with the regulations set forth in the Law on Medical Research Involving Human Subjects and the Good Clinical Practice (GCP) guidelines. This study was approved by the Ethics Committee of the First Affiliated Hospital of Sun Yat-sen University. The relevant document numbers was 45362023653.

## 3 Results

### 3.1 Baseline characteristics

From January 1, 2019, to March 1, 2022, a total of 148 patients with definitive infective endocarditis who underwent surgical therapy with cardiopulmonary bypass were included in our study. The cohort consisted of 110 males and 38 females, with a mean age of 48.2 years. Preoperative blood cultures were negative in 72 cases and positive in 76 cases. Among these patients, 106 did not receive HA380, while 42 received HA380. The period of HA380 use ranged from September 1, 2020 and March 1, 2022. After PSM, 78 patients were evenly distributed into the HA380 group and the non-HA380 group. The baseline characteristics of the patients are detailed in Table 1. The proportion of patients with preoperative lung disease was higher in the HA380 group compared to the non-HA380 group (31% vs. 15.1%, *p* = 0.049). The preoperative albumin level was lower in the HA380 group (34.5 vs. 36.2 g/L, *p* = 0.048). The difference in New York heart association (NYHA) functional class scores between the two groups was statistically significant (HA380 vs. non-HA380, NYHA II 35.8% vs. 40.5%; NYHA III 49.1% vs. 23.8%; NYHA IV 15.1% vs. 35.7%). No other clinical variables showed statistically significant differences. However, after PSM, none of the baseline differences remained statistically significant.

**Table 1:** Preoperative characteristics and surgical information.

Preoperative characteristics and surgical information						
	Unadjusted			After PSM		
	Non-HA380	HA380	*P*-value	Non-HA380	HA380	*P*-value
	(*N* = 106)	(*N* = 42)		(*N* = 39)	(*N* = 39)	
Demographic information						
Age, years						
Mean (SD)	46.9 (15.7)	49.5 (15.0)	0.342	48.1 (16.2)	48.2 (14.7)	0.983
Gender						
Female	26 (24.5%)	12 (28.6%)	0.765	12 (30.8%)	12 (30.8%)	1
Male	80 (75.5%)	30 (71.4%)		27 (69.2%)	27 (69.2%)	
BMI, kg/m^2^						
Mean (SD)	21.1 (4.17)	20.5 (3.45)	0.408	20.3 (3.40)	20.4 (3.40)	0.836
Preoperative comorbidities						
Diabetes						
	10 (9.4%)	8 (19.0%)	0.182	3 (7.7%)	5 (12.8%)	0.709
Pulmonary disease						
	16 (15.1%)	13 (31.0%)	0.0498*	10 (25.6%)	10 (25.6%)	1
Nephropathy						
	8 (7.5%)	5 (11.9%)	0.601	3 (7.7%)	3 (7.7%)	1
Preoperative tracheal intubation						
	5 (4.7%)	5 (11.9%)	0.227	1 (2.6%)	3 (7.7%)	0.608
Preoperative score						
NYHA						
Ⅱ	38 (35.8%)	17 (40.5%)	0.00439*	17 (43.6%)	17 (43.6%)	0.0955
III	52 (49.1%)	10 (23.8%)		17 (43.6%)	10 (25.6%)	
Ⅳ	16 (15.1%)	15 (35.7%)		5 (12.8%)	12 (30.8%)	
Euroscore II						
Mean (SD)	4.09 (3.84)	8.41 (14.1)	0.0571	3.93 (3.92)	6.08 (11.6)	0.278
APACHEII						
Mean (SD)	3.71 (3.19)	5.52 (5.67)	0.0558	3.67 (2.67)	4.72 (5.02)	0.253
Preoperative blood transfusion and medication						
Blood products transfusion preoperative						
	10 (9.4%)	6 (14.3%)	0.573	3 (7.7%)	4 (10.3%)	1
Gamma globulin transfusion preoperative, g						
Mean (SD)	1.37 (8.38)	4.52 (16.4)	0.242	1.28 (6.56)	1.41 (7.34)	0.935
Preoperative culture						
Blood culture						
Gram-positive cocci（exclude staphylococcus）	41 (38.7%)	13 (31.0%)	0.757	13 (33.3%)	13 (33.3%)	0.718
Gram-negative bacilli	3 (2.8%)	2 (4.8%)		2 (5.1%)	2 (5.1%)	
Abiotrophia	2 (1.9%)	2 (4.8%)		0 (0%)	2 (5.1%)	
Staphylococcus	5 (4.7%)	3 (7.1%)		2 (5.1%)	1 (2.6%)	
Resistant organism	2 (1.9%)	2 (4.8%)		1 (2.6%)	2 (5.1%)	
Fungus	1 (0.9%)	0 (0%)		1 (2.6%)	0 (0%)	
Surgical information						
Surgry						
Aortic valve and/or ASD/VSD	29 (27.4%)	11 (26.2%)	0.52	11 (28.2%)	10 (25.6%)	0.788
Mitral valve and/or ASD/VSD	47 (44.3%)	20 (47.6%)		19 (48.7%)	19 (48.7%)	
Tricuspid valve and/or ASD/VSD	4 (3.8%)	4 (9.5%)		2 (5.1%)	4 (10.3%)	
Multiple valves and/or ASD/VSD	24 (22.6%)	7 (16.7%)		6 (15.4%)	6 (15.4%)	
Valve replacement and CABG	2 (1.9%)	0 (0%)		1 (2.6%)	0 (0%)	
Redo						
	8 (7.5%)	2 (4.8%)	0.806	1 (2.6%)	2 (5.1%)	1
Perfusion time, min						
Mean (SD)	159 (74.0)	152 (73.5)	0.601	136 (56.4)	149 (75.2)	0.375
Aortic clamping time, min						
Mean (SD)	94.7 (46.8)	88.7 (45.5)	0.473	80.8 (37.6)	86.5 (46.0)	0.555

EF, ejection fraction; BMI, body mass index; NYHA, New York Heart Association.

### 3.2 Endpoint events

The summary of the endpoint events is presented in Table 2.

**Table 2:** Endpoint information between two groups before and after PSM.

Endpoint						
	Unadjusted			After PSM		
	Non-HA380	HA380	*P-*value	Non-HA380	HA380	*P*-value
	(*N* = 106)	(*N* = 42)		(*N* = 39)	(*N* = 39)	
Sepsis						
	19 (17.9%)	6 (14.3%)	0.772	8 (20.5%)	6 (15.4%)	0.724
Time from operation to discharge days, d						
Mean (SD)	24.6 (13.3)	23.2 (12.6)	0.549	28.1 (14.1)	21.2 (10.3)	**0**.**014**
Length of stay, d						
Mean (SD)	37.5 (16.1)	34.1 (13.5)	0.204	40.3 (18.2)	32.2 (11.0)	**0**.**024**
ICU stay time, h						
Mean (SD)	70.4 (138)	136 (310)	0.193	79.2 (171)	56.3 (81.6)	0.452
Ventilator use time, h						
Mean (SD)	34.9 (82.9)	91.2 (255)	0.168	28.2 (61.8)	29.2 (67.6)	0.944
CRRT						
	11 (10.4%)	9 (21.4%)	0.132	7 (21.9%)	3 (7.7%)	0.289
IABP						
	9 (8.5%)	3 (7.1%)	1	3 (7.7%)	3 (7.7%)	1
ECMO						
	2 (1.9%)	1 (2.4%)	1	2 (5.1%)	1 (2.6%)	1

CRRT, continuous renal replacement therapy; ECMO, extracorporeal membrane oxygenation; IABP, intra-aortic balloon pump therapy.

Bold indicates that the *P*-value is <0.05, and the difference is statistically significant.

#### 3.2.1 Primary outcome

Before PSM, the incidence of postoperative sepsis did not show a statistically significant difference (17.9% vs. 14.3%, *p* = 0.722). After PSM, no statistically significant difference was found between the HA380 group and the control group (15.4% vs. 20.5%, *p* = 0.724).

#### 3.2.2 Secondary outcomes

Before PSM, there were no significant differences in time from operation to discharge (24.6 vs. 23.2 days, *p* = 0.549), length of stay (37.5 vs. 34.1 days, *p* = 0.204), ICU stay time (70.4 vs. 136 h, *p* = 0.193) and Ventilator use time (34.9 vs. 91.2 h, *p* = 0.168). Additionally, there were no differences in the use of CRRT (10.4% vs. 21.4%, *p* = 0.132), IABP (8.5% vs. 7.1%, *p* = 1.0) or ECMO (1.9% vs. 2.4%, *p* = 1.0) therapy between the two groups. After PSM, Time from operation to discharge was shorter in the HA380 group (21.2 vs. 28.1, *p* = 0.014), as well as length of stay (32.2 vs. 40.3, *p* = 0.024). There were no differences in ICU stay time (79.2 vs. 56.3 h, *p* = 0.452) and ventilator use time (28.2 vs. 29.2 h, *p* = 0.994), CRRT (21.9% vs. 7.7%, *p* = 0.289), IABP (7.7% vs. 7.7%, *p* = 1.0) or ECMO (5.1% vs. 2.6%, *p* = 1) therapy between the two groups.

### 3.3 SOFA scores and postoperative laboratory results

**Figure 1:**
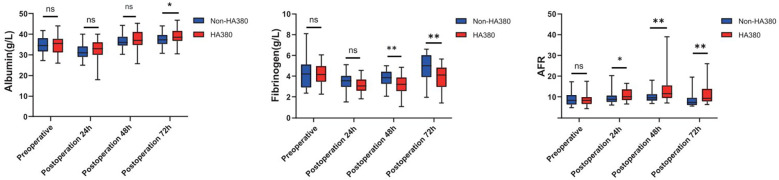
Boxplot of albumin, fibrinogen and AFR throughout the perioperative period.

We collected relevant SOFA scores and postoperative laboratory results of the patients, as shown in Table 3. The difference in postoperative SOFA scores between the two groups was not statistically significant (24 h: 2.97 vs. 1.92, *p* = 0.327; 48 h: 2.28 vs. 1.49, *p* = 0.289; 72 h 1.21 vs. 0.767, *p* = 0.167). In the HA380 group, fibrinogen levels were significantly lower than in the non-HA380 group (24 h: 3.15 vs. 3.51, *p* = 0.053; 48 h: 3.14 vs. 3.82, *p* = 0.002; 72 h: 3.94 vs. 4.80, *p* = 0.006). Conversely, the albumin-to-fibrinogen ratio (AFR) was significantly higher in the HA380 group compared to the non-HA380 group (24 h: 10.9 vs. 9.53, *p* = 0.038; 48 h: 13.4 vs. 10.0, *p* = 0.005; 72 h: 11.1 vs. 8.57, *p* = 0.009) (Figure 1). The differences in other laboratory results were not statistically significant.

**Table 3:** Laboratory examination between two groups before and after PSM.

SOFA score and laboratory examination						
	Unadjusted			After PSM		
	Non-HA380	HA380	*p*-value	Non-HA380	HA380	*p*-value
	(*N* = 106)	(*N* = 42)		(*N* = 39)	(*N* = 39)	
Before surgery						
SOFA						
Mean (SD)	1.58 (2.17)	2.14 (4.43)	0.439	1.00 (1.95)	1.77 (4.15)	0.299
ALT, U/L						
Mean (SD)	33.4 (54.5)	29.0 (23.2)	0.498	24.0 (17.2)	26.4 (16.7)	0.537
ST, U/L						
Mean (SD)	34.6 (45.6)	37.1 (33.9)	0.719	29.4 (19.5)	33.8 (26.6)	0.409
TNT, ug/L						
Mean (SD)	0.829 (4.67)	0.145 (0.214)	0.136	0.645 (2.99)	0.134 (0.211)	0.089
BNP, pg/ml						
Mean (SD)	2,780 (5,420)	4,140 (7,900)	0.312	1,280 (1,380)	3,250 (6,930)	0.089
PCT, ng/ml						
Mean (SD)	2.49 (12.9)	1.10 (1.02)	0.275	1.21 (1.08)	1.11 (1.03)	0.704
WBC, ×10^9^/L						
Mean (SD)	8.94 (3.92)	9.85 (4.80)	0.279	8.59 (3.94)	9.87 (4.93)	0.212
Hb, g/L						
Mean (SD)	109 (21.2)	104 (19.8)	0.174	108 (18.6)	105 (19.9)	0.57
24 h after surgery						
SOFA						
Mean (SD)	2.43 (4.82)	1.86 (3.30)	0.407	2.97 (5.70)	1.92 (3.42)	0.327
ALT, U/L						
Mean (SD)	64.3 (151)	125 (604)	0.524	34.8 (22.3)	132 (627)	0.335
AST, U/L						
Mean (SD)	132 (280)	459 (2,430)	0.389	72.1 (37.3)	489 (2,520)	0.309
TNT, ug/L						
Mean (SD)	1.97 (5.42)	1.52 (1.78)	0.456	1.25 (0.899)	1.56 (1.84)	0.282
BNP, pg/ml						
Mean (SD)	2,520 (6,100)	2,380 (4,130)	0.871	1,500 (3,190)	2,110 (4,090)	0.484
PCT, ng/ml						
Mean (SD)	2.42 (10.9)	0.611 (0.983)	0.094	1.79 (6.62)	0.532 (0.904)	0.24
WBC, ×10^9^/L						
Mean (SD)	15.8 (6.59)	15.1 (6.41)	0.554	14.2 (6.38)	15.4 (6.49)	0.353
Hb, g/L						
Mean (SD)	98.4 (13.2)	95.1 (12.3)	0.157	97.8 (14.1)	95.8 (12.3)	0.524
48 h after surgery						
SOFA						
Mean (SD)	1.91 (3.31)	1.40 (2.67)	0.34	2.28 (3.74)	1.49 (2.75)	0.289
AST, U/L						
Mean (SD)	164 (402)	70.9 (72.4)	**0**.**024**	70.0 (51.5)	72.2 (74.9)	0.876
TNT, ug/L						
Mean (SD)	1.57 (4.23)	1.26 (2.87)	0.612	0.936 (1.64)	1.28 (2.98)	0.529
BNP, pg/ml						
Mean (SD)	4,320 (7,600)	3,760 (4,800)	0.598	3,360 (4,850)	3,510 (4,860)	0.898
PCT, ng/ml						
Mean (SD)	6.00 (18.3)	1.97 (2.04)	**0**.**028**	6.30 (21.9)	1.77 (1.76)	0.199
WBC, ×10^9^/L						
Mean (SD)	14.2 (6.03)	13.3 (5.58)	0.38	13.6 (5.10)	13.5 (5.59)	0.966
Hb, g/L						
Mean (SD)	93.0 (14.2)	91.4 (12.4)	0.496	96.4 (13.5)	91.4 (12.7)	0.091
72 h after surgery						
SOFA						
Mean (SD)	0.96 (1.49)	0.76 (0.75)	0.167	1.21 (1.78)	0.767 (0.77)	0.167
ALT, U/L						
Mean (SD)	69.6 (178)	23.8 (18.6)	**0**.**01**	24.4 (22.3)	24.4 (19.1)	1
AST, U/L						
Mean (SD)	140 (353)	51.7 (50.5)	**0**.**014**	51.1 (40.9)	51.4 (51.9)	0.977
TNT, ug/L						
Mean (SD)	0.926 (1.73)	0.894 (2.30)	0.936	0.628 (0.902)	0.920 (2.39)	0.481
BNP, pg/ml						
Mean (SD)	5,990 (10,400)	4,640 (5,210)	0.295	4,840 (6,420)	4,590 (5,380)	0.859
PCT, ng/ml						
Mean (SD)	110 (581)	39.8 (43.9)	0.222	52.3 (43.3)	42.6 (44.4)	0.328
WBC, ×10^9^/L						
Mean (SD)	17.8 (7.19)	16.4 (6.28)	0.254	17.2 (7.27)	16.7 (6.31)	0.758
Hb, g/L						
Mean (SD)	91.4 (12.9)	88.8 (11.8)	0.235	93.5 (13.8)	89.0 (12.2)	0.109
PLT, ×10^9^/L						
Mean (SD)	138 (60.9)	143 (67.0)	0.667	139 (56.3)	150 (64.2)	0.436

AFR, albumin-fibrinogen ratio; ALT, alanine aminotransferase; AST, aspartate aminotransferase; TNT, troponin T; BNP, B-type natriuretic peptide; PCT, procalcitonin; WBC, white blood cell; HB, hemoglobin; SOFA, sequential organ failure assessment.

Bold indicates that the *P*-value is <0.05, and the difference is statistically significant.

## 4 Discussion

CPB may induce a sudden release of inflammatory cytokines. Theoretically, the application of HA380 could facilitate the adsorption of inflammatory mediators, thereby contributing to improved postoperative outcomes (14, 18). However, in our study, no statistically significant difference in the incidence of sepsis was observed between the HA380 and non-HA380 groups. Additionally, compared to the non-HA380 group, the HA380 group exhibited shorter postoperative hospital stay, shorter total hospital stay, lower fibrinogen level and a higher AFR.

Wang et al. found that although the serum IL-6 levels increased more rapidly in the control group than in the HA380 group after surgery, the incidence of postoperative acute kidney injury(AKI) and acute respiratory distress syndrome(ARDS) was lower in the HA380 group. However, the incidence of other postoperative complications, including ventilation time, ICU stay, hospital stay, and in-hospital mortality were not significantly different between the two groups (14). While in a 2022 study, patients in the HA380 group had significantly lower IL-6 levels, required less vasopressin, had shorter mechanical ventilation duration, and had shorter ICU stays. The authors concluded that HA380 was effective in reducing SIRS and promoting postoperative recovery (19). In studies of blood adsorption using other perfusion devices, several studies have reported no differences in the incidence of sepsis, ICU length of stay, ventilator treatment, and 30-day mortality rate following blood adsorption. Haidari et al. indicated that the sepsis-related mortality rate was lower in patients who underwent blood adsorption (34% vs. 43%, *p* = 0.041), while there were no differences in the incidence of sepsis or in-hospital mortality (11, 20, 21). Our study failed to demonstrate that the use of HA380 could reduce the incidence of postoperative sepsis. Despite shorter hospital stays, there were no differences in incidence of sepsis, ICU length of stay, ventilator use, CRRT, IABP or ECMO.

Blood adsorption effectively lowers fibrinogen levels, as evidenced by a significant reduction observed at the end of the procedure (22).Studies have linked lower fibrinogen levels in sepsis patients to higher mortality rates (23, 24), with thresholds below 1.6 g/L or 2.0 g/L showing a stronger correlation (25, 26). In the early stages of sepsis, fibrinogen levels rise, exacerbating inflammation (27, 28), while albumin levels typically decrease (29), reflecting the complex interplay of inflammation. As a novel biomarker, AFR has shown utility in various conditions, including cancer and autoimmune diseases (30–32). While cytokine adsorption has the potential to regulate immune responses, its clinical advantages remain poorly defined. Our research indicated significant reductions in fibrinogen levels and increases in AFR in the HA380 group; however, these findings did not translate into improved clinical outcomes. Consequently, the clinical significance of these results requires further investigation in the future.

Some reports suggest that propensity score matching (PSM) can reduce or even eliminate the impact of selection bias in both prospective and retrospective studies (33, 34). In this retrospective study, PSM was employed to match baseline characteristics, including demographic data, preoperative complications, medications, laboratory tests, and surgical details, aiming to reduce differences in disease severity and physical condition between groups. However, the matching for patients using HA380 was not strictly adjusted for confounding factors. Despite PSM's partial adjustment, the impact of unknown confounders persists. Additionally, the study did not collect or analyze more cytokines, such as interleukins and interferons, nor did it observe their specific changes.

Our study has several limitations. First, as a single-center retrospective study, it is limited by a relatively small sample size and inherent internal biases. Second, although PSM analysis was employed to balance baseline data, further multicenter, large-sample prospective clinical studies are required to validate our conclusions. Additionally, more detailed designs for laboratory tests should be implemented, such as collecting simultaneous data on inflammatory factors in patients, to better elucidate the specific effects and mechanisms of HA380.

## 5 Conclusion

The use of HA380 in surgical interventions for infective endocarditis did not result in a decreased incidence of postoperative sepsis. Although the HA380 group showed a shorter postoperative hospital stay, shorter total hospital stay, lower fibrinogen level, and a higher AFR, its overall effectiveness still requires further validation.

## Data Availability

The raw data supporting the conclusions of this article will be made available by the authors, without undue reservation.
